# P-1621. Utilization of Repeated Procalcitonin Assays at A Tertiary Care Center

**DOI:** 10.1093/ofid/ofae631.1788

**Published:** 2025-01-29

**Authors:** Reema Andrade, Sarah J Norman, Sheena Ramdeen

**Affiliations:** MedStar Washington Hospital center, Washington DC, Washington DC, District of Columbia; MedStar Washington Hospital Center, Bethesda, Maryland; MedStar Washington Hospital Center, Bethesda, Maryland

## Abstract

**Background:**

Procalcitonin (PCT) is a biomarker which has been shown to be safe and effective for reducing antibiotic exposure in patients with respiratory infections and sepsis. In current clinical practice, PCT has become a promising new biomarker for the early detection of systemic bacterial infections. In 2017, the US Federal Drug Administration (FDA) approved its use to guide antibiotic stewardship efforts in people with these conditions. Prior studies have also found value in repeated PCT measurements to inform antibiotic use in the first few days of empiric treatment. Although the use of PCT has steadily increased, failure to use the results in clinical decision-making limits its utility in antibiotic stewardship. The purpose of this study was to describe how repeated PCT tests are utilized at a large tertiary care center to guide antibiotic stewardship.

**Figure:**
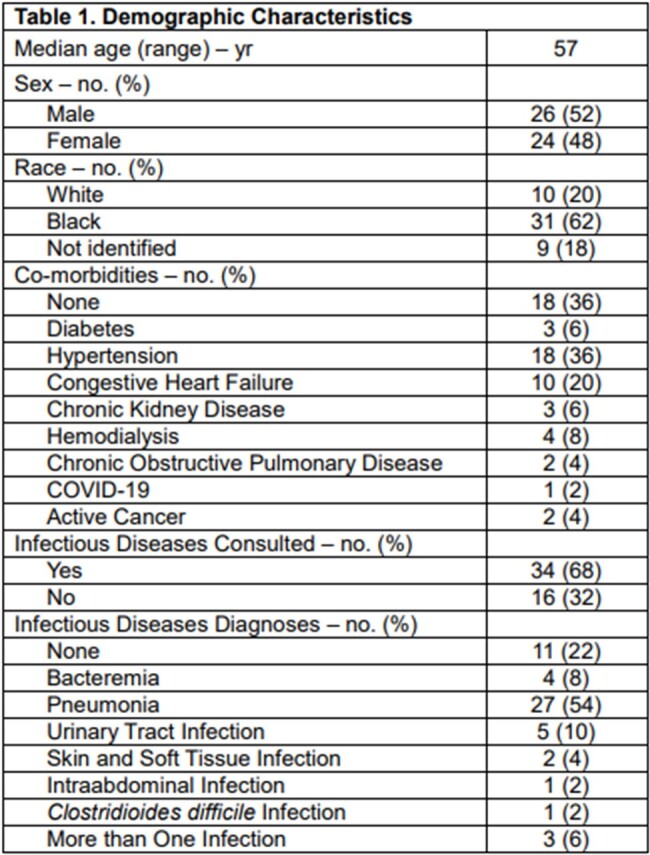

**Methods:**

There were 1,002 patients between April 2023 and October 2023 who had a PCT test ordered, including 116 with two or more tests. Fifty of those with at least two PCT tests were selected randomly for a retrospective chart review and analysis.

**Figure:**
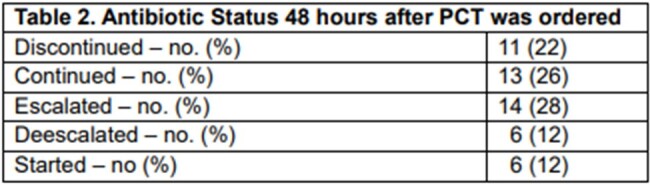

**Results:**

The median age of the cohort was 57 years, with 26 (52%) males and 24 (48%) females. Twenty-six patients received antibiotics before the PCT was tested, and 24 did not. Among the patients with at least two PCT tests, antibiotics were discontinued in 11 (22%), continued in 13 (26%), escalated in 14 (28%), and de-escalated in 6 (12%). Nine (53%) of the patients whose antibiotics were discontinued or de-escalated had PCT values that improved on repeat tests, and eight (43%) had PCT values that increased. The most common infectious diseases diagnosis was pneumonia, and three patients had more than three concurrent infections. Twenty patients had more than two PCT tests ordered. The median length of stay was 33 days, and ten patients died during their hospitalization.

**Conclusion:**

Having more than one PCT test may be helpful in clinical decision-making and guiding antibiotic stewardship efforts, but it did not appear to have a major impact in reducing antibiotic use in this cohort. The study findings support the need for further optimization of PCT testing to aid in antibiotic stewardship at this center.

**Disclosures:**

**All Authors**: No reported disclosures

